# Why We Should Buy Goods Made in U. S. A.

**Published:** 1915-02

**Authors:** John W. Lambert

**Affiliations:** St. Louis, Mo.


### WHY WE SHOULD BUY GOODS MADE IN U. S. A.

BY JOHN W. LAMBERT, ST. LOUIS, MO.

Collier's Weekly is doing commendable work in urging the American people to buy goods "Made in U. S. A." The future welfare of our country and the prosperity of our people depend very largely upon the success of the "Made in U. S. A." propaganda.

Many publications are encouraging the American people to buy American-made goods without giving specific reasons why they should do so. Don't you think that the movement will gain greater impetus and accomplish more for the manufacturer, as well as the consumer, if substantial "reason why" arguments are presented?

You are a student of national problems, while we manufacturers, who are engaged in import and export commerce, are deeply involved in the problems of manufacturing and marketing merchandise. I believe we should work together; therefore I offer you some facts based on my personal experiences in European countries.

With my associates, I am engaged in the manufacture of an article which in consumed in every part of the civilized globe. Our business originated thirty-three years ago and in its entirety is owned by American people. We maintain headquarters, offices and ware-rooms on this continent, also factories in many foreign countries, including France, Germany, Austria and Spain.

Americans and Britishers always have traveled extensively. Years ago they began to demand our product when abroad, and to satisfy this demand we attempted export shipment. Our American factories were then large enough to supply the universal demand, but when entering the foreign field we found it impossible profitably to clear our merchandise in many ports. Germany, Austria, France and Spain demanded prohibitive duties. This left us the choice of discontinuing our export business or yielding to the demands of the foreigner, which in substance were: "If you want to do business in our country, you must pay rents to our landlords, use our raw materials and employ our labor." We were practically forced to equip and maintain factories in the above mentioned places.

Without any desire to criticize the spirit which prompted the demands of these countries, I will say that the disadvantage to us has been very great. But we have had an even greater disadvantage to contend with, to illustrate which I will relate a part of my experience in Germany:

In Berlin I advertised for a highly trained city representative. Many responded and I interviewed and discharged all but two, who were requested to report to me the following Friday. When one man reported he immediately asked for his references, stating that he did not want the position. Upon being questioned he replied about as follows:

"I have spent the last two days interviewing the trade and investigating your product. It is American-owned and the retailers will not push a foreign-owned product. Their customers prefer and demand articles made only in the Fatherland. I see no future for me with your company."

The other man accepted the positon but resigned shortly afterwards. He also found that German people demand goods made in the Fatherland, *by concerns owned in the Fatherland.*

Germany has asked us to buy her goods and we have cheerfully complied with the request. American dollars have made German manufacturers wealthy, yet the German people refuse to buy our goods.

Germany has become a strong nation because her people stick together and work together. They patronize home industry. Nowhere else in the world is the term "home indtistry" understood and appreciated as it is in Germany.

Many foreign-made cosmetics, proprietary medicines, textiles, toys and other articles are sold in America in competition with American-made and owned products that are really as good, and in some cases vastly better, at no higher price than the imported goods. Millions of dollars are expended by American people for French soaps, toilet articles, silks, millinery, gowns, etc. One great Paris firm does a tremendous toilet soap business in America, despite the fact that our domestic manufacturers produce superior soaps at less cost.

Within the last few years foreign manufacturers have established distributing points and even factories on American soil, *but they are foreign-owned.*

When we confine our demand for articles we eat, wear or use to those made in America by American capital and labor, then will our American enterprises grow in leaps and bounds, and since many foreign-owned articles are made in this country, or sold through domestic agents, each article should be carefully scrutinized and *its ownership determined*—so that those which are foreign-owned can be avoided whenever similar products of home manufacture are obtainable.

The German-American press and the agents of the Kaiser are vigorously protesting against the lack of American sympathy for their cause, but perhaps it does not occur to them that our spirit of fair play, even our sense of humor, does not permit us to approve of or enjoy what may be styled a travesty on reciprocity—a burlesque on equity!

Foreign governments have done much to assure tariff protection of their industries, but in some countries it has rested with the people as individuals to do far more than it is possible to accomplish by stringent legislation. True patriotism means 100 per cent. protection.

I am withholding the name of the company in which I am interested for the reason that I am not seeking free publicity for its product. [Listerine. Editor *Register.*]

We like the above letter because it gives real facts, based upon real experience. Americans are a tolerant, easy-going people in business matters, largely because prosperity generally has come to us without great effort. Our commerce of the future must be based upon more conscious effort, more skill in marketing and more patriotic support of our own industries, because more than ever we are going to face the effort, skill and commercial patriotism of foreign nations.

Foreign commerce is crippled now, but when the war is over it will meet us with redoubled effort in every market of the world, particularly our own. Therefore it behooves us to see that the goods we consume, wherever possible, are

MADE IN THE UNITED STATES, AND THAT THEY BEAR THE NAME AND TRADE-MARK OF THE MANUFACTURER AS WELL AS THE NATIONAL TRADE-MARK "MADE IN U. S. A."

—E. C. Patterson, in *Collier's,* for January 16, 1915.

While we have much sympathy with this attitude toward foreign-made goods, we must remember that Germany is a comparatively small country and is supporting a large population and a most expensive Government, and she is compelled to extend her trade relations to every profitable source possible. Her great needs in a measure justify her seeming unfriendly commercial relations. America will need German products for many years to come and can afford to tolerate apparent commercial discourtesies.—Editor, *Register.*

